# High-grade transformation of adenoid cystic carcinoma of parotid gland with isolated intratemporal facial nerve involvement: a case report and literature review

**DOI:** 10.1186/s43046-022-00144-1

**Published:** 2022-09-19

**Authors:** Manu Coimbatore Balakrishnan, Abhijeet Bhatia, Bifica Sofia Lyngdoh, Caleb Harris, Donboklang Lynser, Pranjal Kalita

**Affiliations:** 1 Department of Otorhinolaryngology, North Eastern Indira Gandhi Regional Institute of Health and Medical Sciences, Shillong, 793018 India; 2 Department of Pathology, North Eastern Indira Gandhi Regional Institute of Health and Medical Sciences, Shillong, 793018 India; 3 Department of Surgical Oncology, North Eastern Indira Gandhi Regional Institute of Health and Medical Sciences, Shillong, 793018 India; 4 Department of Radiology, North Eastern Indira Gandhi Regional Institute of Health and Medical Sciences, Shillong, 793018 India

**Keywords:** Carcinoma, Adenoid cystic, Facial paralysis, Parotid gland, Temporal bone, Case report

## Abstract

**Background:**

High-grade transformation Adenoid cystic carcinoma (HGT-AdCC) of the parotid gland is a rare transformation noted in slow growing low grade AdCC. Perineural invasion and spread is an important feature of this tumor. Temporal bone involvement is rare. A total of only 10 cases of HGT-AdCC in parotid gland has been reported in literature so far predominantly in the elderly with peak incidence in 5th–6th decade.

**Case presentation:**

We present a young lady of HGT-AdCC of right parotid gland with temporal bone involvement in the form of isolated perineural invasion (PNI) of facial nerve till the tympanic segment. She underwent right radical parotidectomy with modified radical neck dissection with modified lateral temporal bone resection and pectoralis major myocutaneous flap reconstruction. Histopathological examination revealed both low- and high-grade areas. Sections from facial nerve showed tumor invasion.

**Conclusion:**

The radiological features of isolated perineural spread in intratympanic part of facial nerve can be easily missed if not specifically looked for. Every attempt should be made preoperatively and intraoperatively to determine the complete extent of the tumor for adequate disease clearance. A combined clinico-radiological approach aided by histopathology examination helps in early detection of this carcinoma and in better patient management.

## Background

Adenoid cystic carcinoma (AdCC) constitutes about 1% of all malignant tumors of the oral and maxillofacial region. Two-thirds of the tumor are reported in the minor salivary glands. Only 2–3% of all tumors of the parotid are diagnosed as AdCC [1, 2]. High-grade transformation adenoid cystic carcinoma (HGT-AdCC) of the parotid gland is a rare transformation noted in slow growing low grade AdCC. HGT-AdCC has higher propensity for local recurrence and distant metastasis. Perineural invasion and spread is an important feature of this tumor and is classically considered as an adverse prognostic indicator [3]. The usual extra glandular spread in locally advanced parotid cancer will be to the external ear or temporomandibular joint. Temporal bone involvement is rare. A total of only 10 cases of HGT-AdCC in parotid gland has been reported in literature so far [4].HGT-AdCC is a tumor of the elderly with peak incidence in 5th–6th decade [2, 5]. We present a young lady of HGT-AdCC of right parotid gland with temporal bone involvement in the form of isolated perineural invasion (PNI)of facial nerve.

## Case presentation

**Fig. 1:**
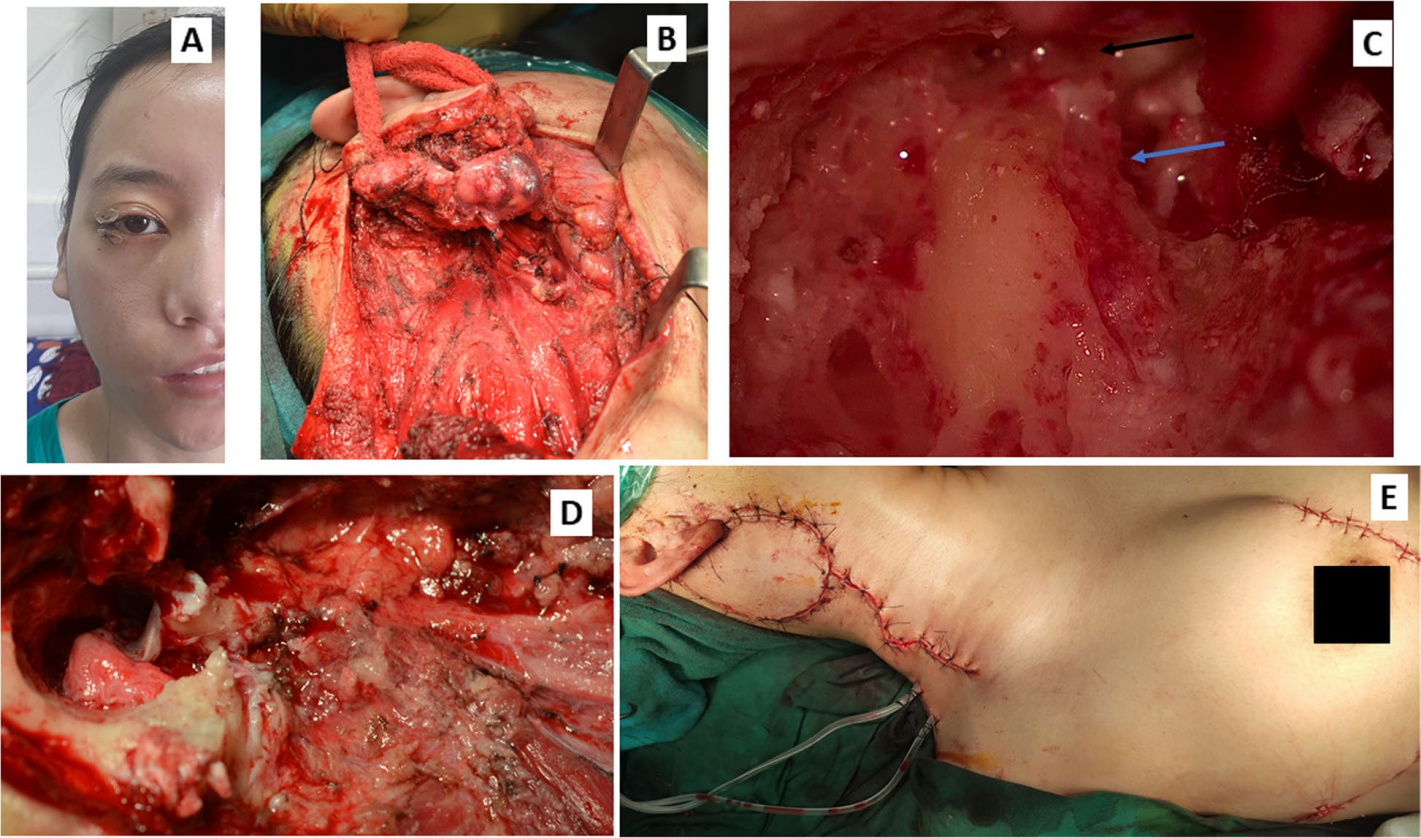
**A** Right facial palsy with lateral tarsorrhaphy. **B** Resected parotid specimen with skin, attached lymph nodes with ligated end of IJV. **C** Blue arrow: involved part of facial nerve at second genu extending 3 mm into the tympanic segment, Black arrow: normal part of tympanic segment. **D** Resected lateral temporal bone, mastoid tip, drilled anterior EAC, surgical bed post excision. **E** Wound closed with PMMC flap

A 28-year-old lady with no significant comorbidities presented with a painful swelling behind right ear for 6 years and facial asymmetry for 1 month. While she was advised surgery by many centers which she visited previously, she refused to undergo surgery as she was apprehensive. Her systemic examination was unremarkable. She had a firm swelling of 7 × 4 cm in neck extending into the infra-aural region with two palpable cervical lymph nodes in level II. There was fixity to the skin and the swelling was not mobile from underlying structures. Neurological examination revealed right facial palsy of grade VI House-Brackmann staging with rest being unremarkable [6]. Rest of the head and neck examination was normal (Fig. 1).

Complete oncological workup had unremarkable blood tests without any evidence of distant metastasis. Contrast enhanced Magnetic resonance imaging scan revealed right parotid gland lesion of size 7 × 6 × 4 cm involving the superficial and deep lobes. There was loss of fat plane with adjacent sternocleidomastoid muscle, subcutaneous tissue, skin and right internal jugular vein. Multiple necrotic enlarged cervical lymph nodes in right levels II and III were noted, largest measuring 3.5 cm.

**Fig. 2:**
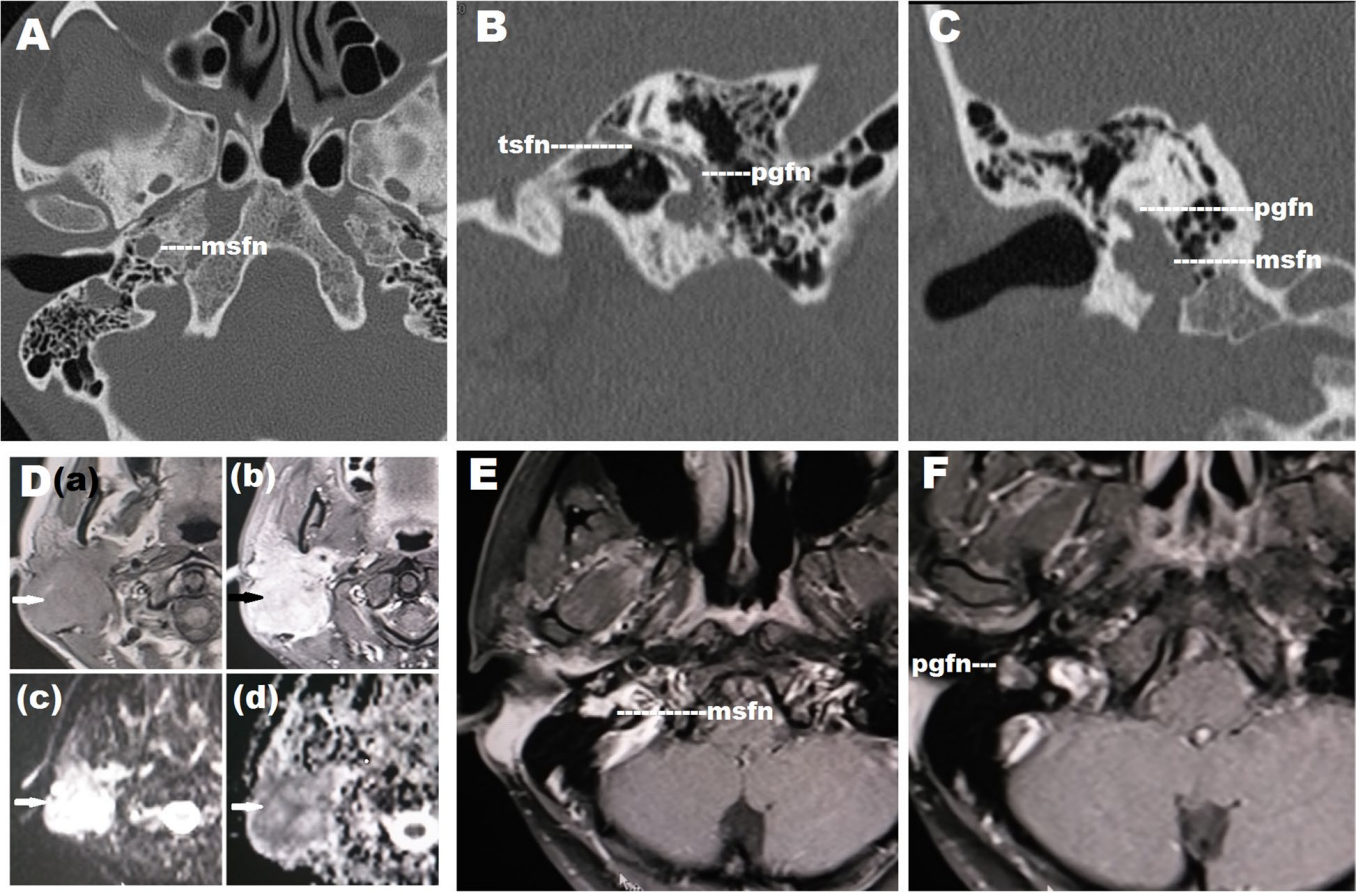
HRCT and CE-MRI findings of adenoid cystic tumor of right parotid (arrow) in a 26-year-old female patient showing perineural extension along the facial nerve. **A** HRCT scan in axial section showing the enlarged canal for mastoid segment of facial nerve (msfn)**. B** HRCT in oblique sagittal section showing enlarged posterior genu of facial nerve (pgfn) with 3 mm extension into tympanic segments (tsfn) of facial nerve. **C** HRCT in oblique coronal section showing enlarged posterior genu (pgfn) and mastoid segments (msfn). **D** (a, b, c, d). (a) Showing the parotid lesion as isointense on T1WI, (b) enhancing on post contrast T1WI, (c) hyperintense on DWI, and (d) hypointense on ADC maps indicating restriction of diffusion. **E** Axial contrast enhanced MRI showing enhancement of mastoid segment of facial nerve (msfn). **F** Axial contrast enhanced MRI showing patchy enhancement with thickening of the posterior genu of facial nerve(pgfn)

High-resolution computed tomography scan of the temporal bone was done specifically to study the temporal bone involvement. It revealed enlargement of the vertical segment of the facial canal from the stylomastoid foramen for a length of approximately 10 mm, extending 3 mm proximal to the second genu into the tympanic segment. There was minimal adjacent erosion with sclerosis in the mastoid segment (Fig. 2).

Initial review of fine needle aspiration cytology (FNAC) slides received from a peripheral institute revealed good cellularity consisting of basaloid cells in 2- and 3-dimensional clusters. FNAC slides showing predominantly basaloid cells is a diagnostic dilemma as it can mimic benign as well as malignant neoplasms [7]. However, based on clinical suspicion and concomitant radiological findings a repeat FNAC was done which showed small to medium sized cells in clusters, acinar and papillary patterns. These cells had hyperchromatic nuclei with moderate amount of cytoplasm. Background showed bare nuclei along with cyst macrophages. These findings were favoring a differential diagnosis of Polymorphous low-grade adenocarcinoma (PLGA), papillary cystic acinic cell carcinoma or AdCC.

Hence, a preoperative diagnosis of parotid carcinomacT4_a_N_2b_M_0_ was made. In view of right lower motor neuron type facial palsy, she underwent right lateral tarsorrhaphy preoperatively. She then underwent right radical parotidectomy with modified radical neck dissection (MRND) preserving spinal accessory nerve with modified lateral temporal bone resection (LTBR) and pectoralis major myocutaneous flap (PMMC) reconstruction under general anesthesia. Intraoperatively parotid tumor of size 5 × 4 cm was noted involving the skin, upper part of sternocleidomastoid muscle, external carotid artery distal to lingual artery and internal jugular vein. These structures were sacrificed and removed along with the specimen. The tumor was also seen to involve the temporal bone via the stylomastoid foramen. Facial nerve canal was decompressed from I genu till the stylomastoid foramen. It was noted that the tumor was having retrograde perineural spread via the stylomastoid foramen involving the intratemporal facial nerve up to 3 mm beyond the II genu, into the tympanic segment. There was widening of the facial canal without extension into the surrounding area of the temporal bone. The tumor was also seen to involve jugular fossa. Resection of facial nerve was done at the tympanic segment proximal part. Intraoperatively frozen section was done to ensure free margin of the nerve. The resected facial nerve was removed with the main specimen and the facial canal was drilled to ensure complete removal of the tumor. En-bloc removal of the parotid tumor with mastoid tip was done. Postoperative period was uneventful.

**Fig. 3:**
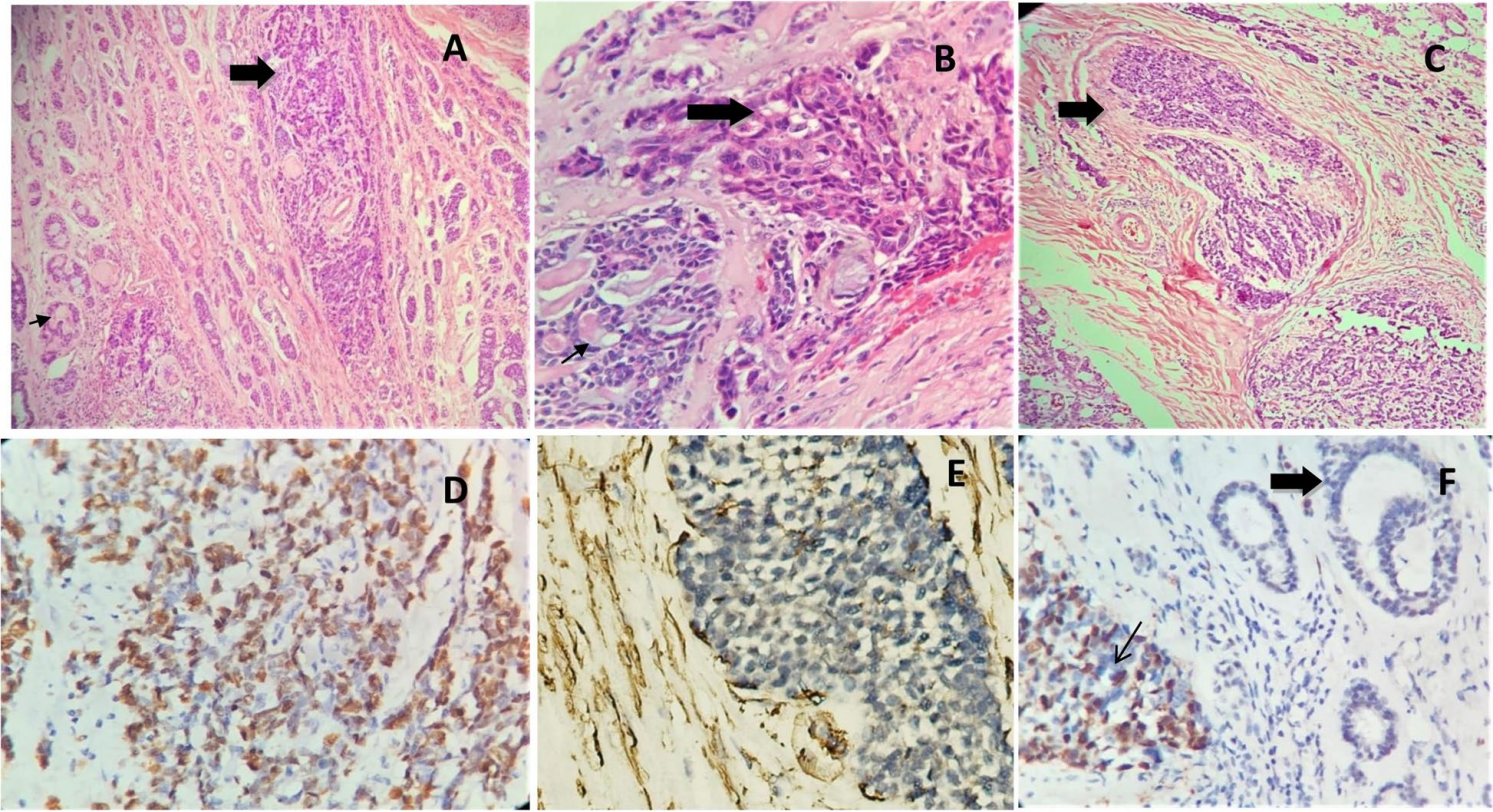
Histopathological examination and immunohistochemistry studies of the tumor. The tubular and cribiform pattern of conventional ACC (thin arrow) with a transition to a solid high grade carcinoma (thick arrow) **A**) H&E x100, **B**) H&E x400. **C**) Tumour infiltrating a nerve (thick arrow) H&E x100. Ki67 labelling index was **D**) 70% in High grade component. Smooth muscle actin (SMA) showed **E**) Loss of expression in high grade component. p53 was **F**) strongly positive in the high grade components (thin arrow) and weakly positive/negative in the low grade components (thick arrow)

Histopathological examination revealed both high-grade and low-grade areas. Low-grade areas showed predominant cribriform architecture whereas the high-grade areas showed mainly solid architecture (45%) with increased mitosis (14/10 hpf), necrosis, cellular atypia and desmoplastic stroma. Sections from facial nerve showed tumor invasion. A total of 7 out of 19 lymph nodes showed tumor involvement (pT_4a_N_3b_M_0_) (Fig. 3). Immunohistochemistry (IHC) studies done on both low- and high-grade areas are shown in Table 1. The patient was later planned for adjuvant radiotherapy. However, the patient refused to undergo any adjuvant therapy due to personal reasons. At 3 months follow-up local recurrence was suspected due to facial swelling and she was advised CEMRI. She refused any further treatment. The radiological and pathological investigations for possible recurrence and metastasis could not be done. On follow-up, it was found the patient died at home 5 months after surgery.

**Table 1:** Immunohistochemistry study of different areas of the tumor

Antibody	Low-grade area	High-grade areas
Ki 67	20 %	65–70%
p53	Weak positivity	Strong positivity
SMA (smooth muscle actin)	Positive	Negative

## Discussion

HGT-AdCC, a rare entity is considered to be a disease of the elderly with male preponderance (Male to female ratio of 1.75:1) [8]. The first case of HGT-AdCC was described by Malhotra et al. in 2009 in an elderly male [9]. Seethala et al. noted the mean age of presentation for HGT-AdCC to be 61 years [8]. HGT-AdCC is a highly aggressive salivary gland neoplasm with high recurrence and poor prognosis [9]. Our case report differs from the previous observation of HGT-AdCC as an entity confined primarily to elderly males and also notes that parotid gland can be a site for primary HGT-AdCC. The overall 5-year and 10-year survival rates for low-grade AdCC ranges from 35-75 % and approximately 20% respectively. The 10-year survival rate for HGT-AdCC is 0%. In contrast, the overall 5-year and 10-year survival rate for the most common carcinoma of parotid gland, i.e., low-grade mucoepidermoid carcinoma is 75–95% and 50% respectively [10, 11]. The factors which affect the prognosis of AdCC of the parotid gland are the presence of facial nerve palsy, perineural spread, temporal bone involvement, histological type, nodal metastasis, distant metastasis, and recurrence [12, 13]. Facial nerve palsy can be seen in up to 70% of patients [14]. It results in poor overall and disease-free survival with a 5-year survival rate of 40%. PNI even if asymptomatic was earlier thought to be a worse overall prognostic indicator, however recent studies indicate that it is a marker of aggressive parotid malignancy and may not necessarily mean poor overall survival [15].

No large sample studies have been done on the survival rates of AdCC of parotid gland with temporal bone involvement. The occult perineural spread if undiagnosed preoperatively can lead to inadequate disease clearance and local recurrence. In a retrospective study over 20 years the incidence of parotid cancer with temporal bone involvement was only 1.1% (30 patients) out of 2700 parotid surgeries. In that case series, the parotid cancers to have occult spread to temporal bone facial nerve in decreasing order were adenocarcinoma (27%), squamous cell carcinoma (23%), salivary ductal carcinoma (14%), and AdCC (13%) with AdCC being one among the least commo n[16]. The commonly described pathway is via stylomastoid foramen with the perineural spread ascending into the vertical segment of facial nerve. In such cases, the temporal bone will show destruction pattern with other extensive local infiltration. This can be demonstrated on CT scan with erosion or widening of the foramen and canal [17]. In our case, the temporal bone showed isolated widening of the facial canal without any gross bony destruction hence this isolated finding could have been easily overlooked as the involvement of facial nerve in intratemporal route by malignant tumors arising from the extratemporal site is rare.

In a previous study of 16 patients done by Leonetti et al. the 5-year and 10-year disease free survival rates of AdCC with temporal bone invasion were 75% and 50% respectively [5]. These relatively high survival rates in this retrospective case series may be attributed to the absence of regional or distant metastasis on presentation and probable low-grade tumor coupled with aggressive therapy. Our patient had HGT-AdCC with temporal bone involvement and regional metastasis.

It has been described that cervical lymph node metastasis is rarely described and that distant metastasis is seen in up to 40% of patients [5]. This was in contrast to our case.

A three tired histopathological grading system for AdCC developed by Perzin and Szanto based on the percentage (%) of solid component [18, 19]. Presence of solid component in an AdCC is a poor prognostic factor. HGT-AdCC or dedifferentiated AdCC is defined as a poorly differentiated adenocarcinoma or high-grade carcinoma that has transformed from a low-grade malignant neoplasm. Histopathological findings with nuclear atypia, increased proliferative index, presence of necrosis and loss of myoepithelial cell, increased Ki 67 index on IHC can help in differentiating it from conventional low grade AdCC [8].

Table 2 depicts the patient demographic features, treatment, follow-up, and outcome of few other studies related to HGT-AdCC of parotid gland.

**Table 2:** Features of patients of HGT-AdCC of parotid gland

Study	Year	Age in years/gender	Diagnosis	Treatment	Outcome
Current study	2022	28/Female	HGT-AdCC Parotid	Radical Parotidectomy, MRND, Modified LTBR, PMMC flap reconstruction. (patient refused adjuvant radiotherapy)	Expired at 5 months
Hruduka et al. [4]	2020	46/Female	HGT-AdCC Parotid	Superficial parotidectomy, selective neck dissection	Unknown
Ly et al. [22]	2013	88/Female	HGT-AdCC Parotid	Superficial parotidectomy, adjuvant radiotherapy	Alive at 12 months
Bonfitto et al. [23]	2010	49/Female	HGT-AdCC Parotid	Surgical excision, RT	Alive at 33 months follow-up
Boland et al. [24]	2012	45/Male	HGT-AdCC Parotid	Unknown	Alive at 25 months
		52/Female	HGT-AdCC Parotid	Unknown	Died due to other etiology
		61/Female	HGT-AdCC Parotid	Unknown	Alive at 169 months
		56/Male	HGT-AdCC Parotid	Unknown	Alive at 77 months
Malhotra et al. [9]	2009	54/Male	HGT-AdCC Parotid	Parotidectomy, adjuvant radiotherapy	Alive at 5 months follow-up

The notorious perineural spread can be seen at either macroscopic or macroscopic level or bot h[20]. In our case, it was seen at both levels. Perineural invasion is a complex process and the complete mechanism is still unknown. At the molecular level, there is a complicated interaction between several chemokines, glial cell-line derived neurotropic factors, Nerve growth factors, matrix metalloproteinases, tight connections of perineural cells, Schwann cells which in the end results in perineural invasion of the tumor [21].

Despite the poorly understood mechanism, patients with perineural invasion by cancer should undergo extensive surgery. Our patient had perineural spread till the tympanic segment of facial nerve and she underwent extensive surgery including modified LTBR. Such extensive surgery is required to improve the long-term outcome [5, 16].

## Conclusion

HGT-AdCC of the parotid gland in young female is a rare tumor and can present with isolated temporal bone involvement. The radiological features of isolated perineural spread in intratympanic part of facial nerve can be easily missed if not specifically looked for. This can lead to inadequate disease clearance, disease recurrence and treatment failure. Hence, the author would like to emphasize that every attempt should be made preoperatively and intraoperatively to determine the complete extent of the tumor for adequate disease clearance. A combined clinico-radiological approach aided by histopathology and IHC studies help in early detection of this malignant neoplasm and in better patient management. Adjuvant radiotherapy and careful postoperative follow-up is of paramount importance in such patients.

## Data Availability

All data collected and generated are published in this article.

## Abbreviations

AdCC: Adenoid cystic carcinoma
HGT-AdCC: High-grade transformation adenoid cystic carcinoma
PNI: Perineural invasion
FNAC: Fine needle aspiration cytology
MRND: Modified radical neck dissection
LTBR: Lateral temporal bone resection
PMMC: Pectoralis major myocutaneous flap
IHC: Immunohistochemistry
